# The Inferiority of Our Coffee

**Published:** 1870-01

**Authors:** 


### The Inferiority of our Coffee.

A correspondent writing from England says:

My attention has been drawn to an article in a leading daily paper, expatiating on the great inferiority of coffee used by us as a beverage, when compared with its excellence in some other countries, and the writer fortifies his remarks by observing that "a cup of good coffee is as rare in England as a blue diamond.

Without going into the entire analysis, untold adulterations and general treatment of coffee in these islands, which might be too long for insertion, a few remarks on its proper and improper torrefaction must not be regarded as offering any extenuation, or in any degree accounting for, the very inferior article supplied us in the ground state, under the name of coffee, but to inform those who may be desirous of preparing their own, and to induce scientific men to direct their attention to this most important subject.

That to all who have visited the continent, a cup of good coffee is as rare in England as a blue diamond, is an incontrovertible fact, for although the finest coffee the world produces is brought here, we English if ever we procure the choicest quality, and grind our own, have but a vague conception what a cup of good coffee really is; hence, it is evident some "radical defect" must, as has been stated, underlie our mode of preparing the berry. Considering the vast importance of this article, socially and commercially, and the great strides science has recently made, it appears incredible the cause of this defect has never been thoroughly investigated, and an improvement in its preparation suggested by one or other of our great analytical chemists. At the first blush it would appear to be a grave reflection on our wives, cooks and housekeepers, but a very little scientific elucidation will dispel any such idea.

The coffee berry is roasted in this country not only in comparatively close iron cylinders, but also in large quantities at the time; by which means the grosser elements, instead of being evolved and evaporated, become condensed, and from the chemical action of the chlorogenate of potassa upon the cellulose or woody fibre, the generation of a new and most acrid bitter substance, called tannin, is formed (*never to be*

*found in its natural state*), which completely deprives coffee of its *true* flavour.

The method adopted by the natives of Ceylon, recently referred to, "who only take the quantity of berries required for present use and roast them in an *earthen* pot, etc.," though simple, is the only compatible and philosophical process; for by preparing coffee in small quantities, in *open* vessels, all the mineral azotised substances are freely evaporated, and the formation of tannin entirely prevented, whereby the caffeine is retained in its pristine purity.

Now caffeine being to coffee what theine is to tea—the primary principle—its complete preservation should be our cheif aim; consequently the infinate superiority resulting from this primitive mode of treating the berry in Ceylon and other Oriental countries where labor is cheap, at once explains our "radical defect," and reveals the true secret of its more exhilarating properties and full aromatic flavor.

From the foregoing deductions, it is but too manifest that until the hand of science devises some means of preparing the berry in perfect accordance with its constituents, and in quantities commensurate to the requirements of a commercial community, *coffee as it ought to be* must ever remain with the multitude, "as rare as blue diamonds."

NATURAL STEAM, HOT AND COLD BATHS.—The natural bathing facilities at the California Geysers make the locality one of the most popular resorts on the Pacific coast. The ordinary bathing establishment is a feature in its way. It is primitive in its construction, being built of rough boards, and portions of it covered with gunny sacks, yet contains natural steam, which rises from the earth and fills the room, so that upon first entering it the heat and steam are almost unbearable; but in a few seconds become quite comfortable, and in a short time are luxurious. The bather, having steamed himself sufficiently, two steps brings him beneath a stream of hot water, which pours through the roof in a continual volume, and is of just bearable heat. Having steamed and boiled, the bather steps a few feet and plunges into the running brook, which is waist deep, with pure water, cool and fresh from the mountains above. The effect of this course of bathing is to leave the bather refreshed and invigorated in a wonderful degree. Separate establishments are erected for ladies, and with the same facilities.—*Med. and Surg. Reporter.*

